# Quantification of skeletal muscle density, mass and fat fraction using single-energy computed tomography

**DOI:** 10.1016/j.jot.2026.101094

**Published:** 2026-05-07

**Authors:** Jonathan Bammessel, Stefan Bartenschlager, Oliver Chaudry, Nicolai Krekiehn, Fjola Johannesdottir, Ling Wang, Michael Uder, Georg Schett, Klaus Engelke

**Affiliations:** a Department of Medicine 3, Friedrich-Alexander-Universität Erlangen-Nürnberg and Universitätsklinikum Erlangen, Ulmenweg 18, 91054, Erlangen, Germany; b Institute of Radiology, Friedrich-Alexander-Universität Erlangen-Nürnberg and Universitätsklinikum Erlangen, Maximiliansplatz 3, 91054, Erlangen, Germany; c Section Biomedical Imaging, Department of Radiology and Neuroradiology, Kiel University, Kiel, Germany; d Center for Advanced Orthopedic Studies, Beth Israel Deaconess Medical Center, Boston, MA, USA; e Harvard Medical School, Boston, USA; f Department of Radiology, Beijing Jishuitan Hospital, Capital Medical University, National Center for Orthopaedics, Beijing, 100035, PR China; g Institute of Medical Physics (IMP), Friedrich-Alexander-Universität Erlangen-Nürnberg (FAU), Henkestr. 91, 91052, Erlangen, Germany

**Keywords:** Calibration, Computed tomography, Muscle fat fraction, Muscle density, Muscle tissue density, Paraspinal muscle

## Abstract

**Background:**

Currently, CT muscle density is measured in Hounsfield units and is not converted to g/cm^3^. Muscle mass and fat fraction (FF) cannot be measured from CT images.

**Methods:**

We propose a phantomless calibration method to calculate muscle tissue and muscle density in g/cm^3^, as well as muscle FF in percent, based on the measurement of water-offset corrected CT values of muscle and subcutaneous adipose tissue (SAT). Calibration requires the mass density and FF of standard muscle tissue (SMT), as well as the FF of SAT. In this study, the International Commission on Radiation Units (ICRU) definition of muscle tissue with a mass density of 1.05 g/cm^3^ was used as standard muscle tissue (SMT). The CT values of SMT were measured using a phantom scanned with seven different CT scanners employing tube voltages ranging from 80 to 140 kV. Existing CT scans of 42 men aged 72 years or over were used to estimate the accuracy errors of the calibrated parameters of the paraspinal muscles. MR Dixon data from 31 subjects were available for comparing CT and MRI FF.

**Results:**

The mean SMT CT value of the phantom measurements was 47.7 ± 1.9 HU. In line with previous publications, the SMT fat fraction was set to 3%, and the subcutaneous adipose tissue (SAT) FF to 85%. Using an SMT CT value of 48 HU, the mean muscle tissue density, muscle density, and muscle fat fraction (FF) of the 41 subjects were 0.94 ± 0.08 g/cm^3^, 1.04 ± 0.01 g/cm^3^, and 11.7 ± 6.4%, respectively. Simulated variations in SMT density of ±0.02 g/cm^3^, SMT CT value of ±2 HU, SMT FF of 5%, and SAT FF of ±3 HU resulted in relative changes in muscle and muscle tissue densities and in absolute changes in muscle FF of below 5%. Simulated accuracy errors were comparable to those caused by an error of ±5 HU in the water offset corrections of the measured CT values of muscle and SAT. The mean MRI fat fraction values were 4.1% higher than the mean CT FF values. The two measurements exhibited a high degree of linear correlation (r^2^ = 0.88, p < 0.001).

**Conclusions:**

The proposed methodology for muscle calibration in single-energy CT images showed a high degree of agreement with MR Dixon FF measurements. The simulated accuracy errors were comparable to those caused by missing water offset corrections of the measured CT values. This is a proof-of-concept study, further validation in subjects with higher muscle FF and in other muscle groups is required.

**The translational potential of this article:**

Quantitative assessments of muscle properties such as density and fat infiltration are important biomarkers for myopathies, sarcopenia, obesity and potentially for osteoporosis. While MRI techniques are state-of-the-art, the opportunistic use of existing CT scans can support screening strategies and may help to identify more subjects at early risk for muscle and perhaps even bone loss. New CT technology, such as photon counting CT, which reduces radiation exposure by around 50% compared to standard CT, may also make CT attractive for dedicated muscle imaging.

## Graphical abstract

**Figure:**
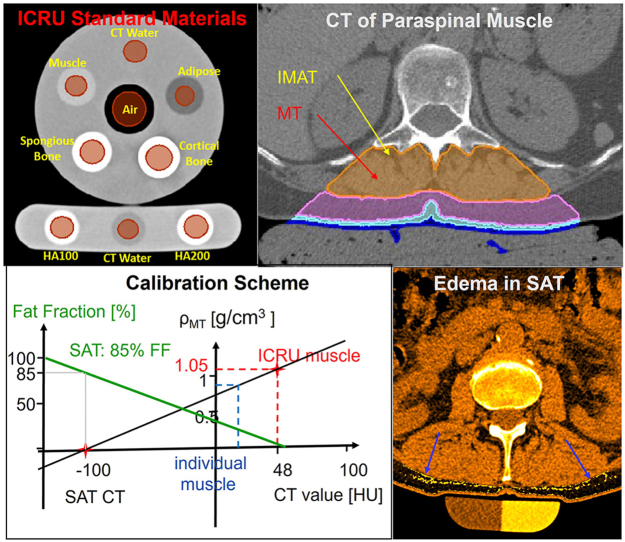

## 1 Introduction

Computed tomography (CT) has been widely used to quantify muscle size and density. However, the advent of Dixon sequences in magnetic resonance imaging (MRI) has established MRI as the gold standard for quantifying muscle fat infiltration, also known as myosteatosis [[1], [2], [3]]. More recently, the use of existing CT scans, called opportunistic screening, to retrospectively detect vertebral fractures, quantify bone mineral density, and determine muscle characteristics has renewed interest in CT for muscle imaging [[4], [5], [6]]. CT has several advantages over MRI, including speed, accessibility, and cost-effectiveness. The use of photon-counting CT technology has the potential to reduce radiation exposure by at least 50% [7].

Currently, CT muscle density is quantified as CT value measured in Hounsfield units (HU), as opposed to physical density, which is measured in g/cm^3^. Muscle CT values are used as cut-off points to assess diseases such as cancer and malnutrition but CT values of these cut-off points vary across studies [[8], [9], [10]]. Also, CT based muscle mass is currently not measured in kilograms, instead muscle cross-sectional area (CSA) measured in cm^2^, or the so-called skeletal muscle index (SMI = CSA/body height^2^) measured in cm^2^/m^2^ are used as surrogates [11]. Alternatively, muscle mass is calculated from the segmented muscle area or volume, assuming a muscle density of 1.04 g/cm^3^ [12]. However, this results in an overestimation of muscle mass because muscle fat infiltration is neglected. CT assessment of fat infiltration usually involves measuring the area of intermuscular adipose tissue (IMAT) or muscle attenuation in HU [11], which differs from fat infiltration measured as a percentage by MRI.

CT values exhibit a linear relationship with density, and muscle density demonstrates a linear relationship with fat infiltration. Consequently, fat infiltration can be assessed through the linear calibration of CT values to fat fraction (FF). A similar issue arises in the calibration of CT values to bone mineral density (BMD). This can be resolved by using a so-called calibration phantom containing reference materials for bone and water, which is scanned simultaneously with the subject. However, analogous phantoms for simultaneous muscle calibration have not been used in clinical settings, and may not exist at all.

In this study, we propose a new phantomless method for quantifying muscle density and FF from measured CT values. The concept of muscle calibration will be described followed by an introduction of the model. The model's assumptions will be discussed in detail. The new calibration technique will be applied to paraspinal muscles using existing CT data of the lumbar spine. This data will also be used to simulate accuracy errors. Finally, the calibrated fat fraction values will be compared to those obtained from MRI scans in a subset of the same subjects.

## 2 Material and methods

### 2.1 Concepts of CT muscle calibration

Muscle as an organ is composed of muscle tissue (MT), which consists of bundles of muscle fibers surrounded by connective tissue layers of fascia. The lipid droplets found within muscle cells are known as intramyocellular lipids (IMCL), while the aggregates of adipocytes found between muscle fibers are referred to as extramyocellular lipids (EMCL). Further adipose tissue is located between muscle fiber bundles. This combination of EMCLs and adipose tissue between muscle bundles is commonly referred to as intramuscular adipose tissue (IMAT = adipose tissue within the muscle organ).

**Fig. 1:**
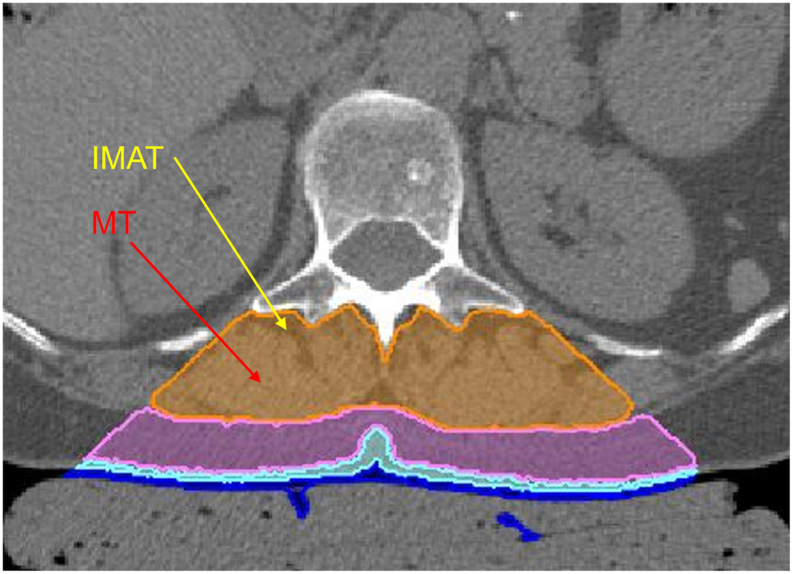
MIAF Volumes of interest: paraspinal muscle (orange), SAT (violet), dermis (light blue) and air (dark blue); IMAT: Intermuscular adipose tissue, MR: muscle tissue

Depending on their function and location, three types of muscle can be distinguished - skeletal, cardiac, and smooth. In a CT (Fig. 1) or MR image (Fig. A4) of skeletal muscle, such as the erector spinae, MT and IMAT can be visually distinguished, although due to the limited spatial resolution, smaller aggregations of adipose tissue will not be visible.

In young, healthy subjects, skeletal muscles consist almost exclusively of muscle tissue, with a fat fraction of around 3-5%, i.e. IMAT is almost invisible in CT or MR images. With increasing age, muscle fat infiltration increases — the amount of MT decreases and IMAT increases. Even the fat fraction of what appears to be MT (black in Fig. A4) increases compared to that of MT in young subjects.

It is important to distinguish between the muscle (M; shown in orange in Fig. 1) and the muscle tissue (MT) within it. The CT value of M is a measure of the amount of MT. If MT is completely replaced by IMAT, the CT value of M will be close to −100 HU. The proposed method calibrates the CT value measured for the whole muscle to the muscle tissue density (ρ_TM_), i.e. the amount of MT in grams within the whole muscle volume. The density of the whole muscle, ρ_M_, can also be determined separately from ρ_TM_. For example, if the muscle tissue is completely replaced by IMAT, then ρ_TM_ is zero and ρ_M_ is equal to the IMAT density.

Analogous to the calculation of BMD in quantitative CT (QCT), the conversion of HU to muscle density in g/cm^3^ can be based on two so called reference materials with known densities [13]. In the case of BMD these are hydroxyapatite and water. In the proposed muscle calibration method, a standard MT and subcutaneous adipose tissue (SAT) serving as a substitute for AT will be used. In QCT, an external calibration phantom containing the reference materials, hydroxyapatite and water is scanned simultaneously with the subject. However, the proposed muscle density calibration method should work without a phantom. This can be achieved if the CT values of the reference materials can be measured in the subject's CT scan or if a universal reference standard can be specified. For such a universal standard CT values must not depend on the type of CT scanner or imaging protocol used, e.g. the x-ray tube voltage.

In the proposed muscle density calibration method, the CT value of SAT is determined in the scan of the subject. Apart from edema, the accumulation of excess fluid with water-like X-ray absorption characteristics, the composition of SAT is rather constant across subjects [14,15]. In contrast, the composition of skeletal muscle tissue found in the human body varies among different muscle groups and locations, as well as with ageing, sex, and disease. Therefore, it is proposed that the specific adult skeletal muscle material, described in the reports of the International Commission on Radiation Units (ICRU) [16,17] be used as the muscle tissue reference. The ICRU adult skeletal muscle material is representative of a muscle of a young healthy subject. The mass and chemical properties of ‘ICRU skeletal muscle, referred to in this study as standard muscle tissue (SMT), are well documented. The ICRU reports list the atomic composition, mass density, and effective atomic numbers, and NIST has published the X-ray absorption coefficients [18].

### 2.2 Proposed muscle calibration model

The following assumptions have been made in order to calibrate CT values to muscle tissue density ρ_MT_ Once ρ_MT_ is known, the muscle density ρ_M,_ in g/cm^3^ and muscle FF in percentage terms can also be derived. (1) The mass density of SMT is 1.05 g/cm^3^ as specified in the ICRU reports. (2) The CT value of SMT is 48 HU. This will be detailed in section 4.3. (3) The composition and therefore the mean CT value of SAT corrected for potential edema is not subject-specific, although it depends on the CT imaging protocol, such as X-ray tube voltage. This will be further discussed in Section 4.5. (4) 85% of SAT consists of fat and 15% consists of fat-free components [14,15]. (5) The FF of IMAT, which like SAT is a white AT was assumed to be also 85% [15]. (6) The mass density of AT is 0.95 mg/cm^3^ as specified by the ICRU [16,17]. (7) SMT also contains some fat, for example ICMLs. For the purposes of this study, a 3% FF is used for SMT based on a retrospective analysis of 6 pt Dixon images from 21 young and healthy subjects (29 ± 5 years) using baseline data from an earlier exercise trial [19]. Average MT FF in those young healthy subjects was 2.7% ± 0.7%.

**Fig. 2:**
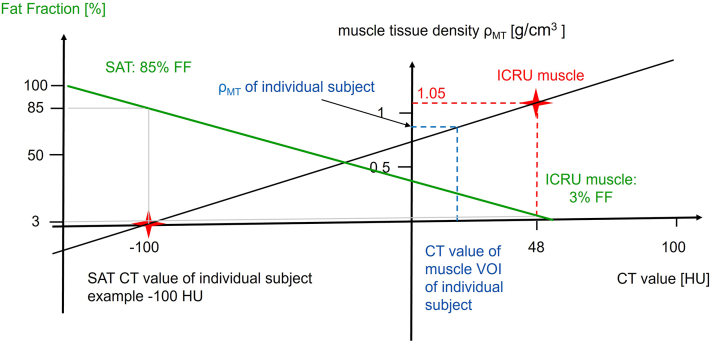
Linear calibration of muscle density in g/cm^3^ (black line) and of muscle fat fraction (FF) in % (green line). The black line for muscle density calibration is defined by the mass density (ICRU value of 1.05 g/cm^3^) and CT value (48HU) of the standard muscle tissue and the SAT CT value of the given scan. The green line for FF calibration is defined by a FF of 3% and density of 1.05 g/cm^3^ of the standard muscle tissue and a FF of 85% of SAT.

The calibration model is graphically illustrated in Fig. 2. Details of the calculations are provided in the Appendix. The results are ρ_MT_ and ρ_M_, both in g/cm^3^ as well as muscle FF in %. Fig. 2 depicts the calibration of ρ_MT_ and of FF. ρ_M_ can then be easily obtained as shown in the Appendix. If the measured CT value of the muscle is 48 HU, then according to the assumptions above the muscle consists of 100% SMT and 0% IMAT, its density is 1.05 g/cm^3^ and the muscle FF is 3%. As fat infiltration increases, the amount of SMT in the muscle decreases as does its density. In Duchenne muscular dystrophy, for example, a muscle may be completely replaced by adipose tissue, which according to the above assumptions, has an FF of 85%.

Deviations from each of the seven assumptions will lead to accuracy errors of muscle density and FF. This will be addressed further in Section 4.6.

### 2.3 CT values of muscle and adipose tissue

To determine the accuracy of using a standard CT value for SMT, an electron density phantom (EDP, QRM, Moehrendorf, Germany) was measured using three different Siemens Somatom scanners (Force, X.cite, go.Top, Siemens Healthcare, Germany) at the University Hospital Erlangen, Germany, on a Philips ICT SP scanner at the University Hospital Kiel, Germany, and on a GE Optima 670, a Philips Icon Spectral, and a Toshiba Aquilion Prime scanner at the Beijing Jishuitan Hospital in Beijing, China. The EDP contains cylindrical inserts of SMT and AT compositions, as specified in ICRU reports 44 and 46 [16,17].

The EDP was scanned on a QRM bone density calibration (BDC) phantom (Fig. A1) at tube voltages ranging from 80 to 140 kV. The sole purpose of the BDC was to determine the CT value of water. In each instance, the EDP was positioned at the center of the gantry. Measurements were performed with 100 mAs with a reconstructed slice thickness of 3 mm, or with 200 mAs with a slice thickness of 1 mm. For reconstruction, a medium-sharp kernel was used. The CT value of the BDC water insert was used to correct for a potential offset of the routine CT scanner calibration. CT values of SMT and AT were compared between scanners and tube voltage settings, both prior to and following water offset correction.

### 2.4 Subjects and CT scans

Baseline CT scans of the lumbar spine, which were previously obtained for the Franconian Osteopenia and Sarcopenia Trial (FrOST) [20] were used for muscle calibration. No subject data were specifically obtained for the analysis presented here. In brief, existing CT scans of 42 community-dwelling men with osteosarcopenia [21], 72 years and older, were reanalyzed. The FrOST study was approved by the Institutional Review Board (IRB) of the University Hospital Erlangen (number 67_15b and 4464b) and the Federal Bureau of Radiation Protection (BfS, number Z 5 - 2246212 - 2017-002).

All subjects’ CT scans were obtained using a Somatom Force scanner (Siemens, Erlangen, Germany; 120 kV, 100 reference mAs, CAREDose 3D, pitch 1, slice thickness and reconstruction increment 1 mm each, BR40s kernel, field of view of 20 cm). Subjects were scanned on top of a Siemens Osteo calibration phantom used for BMD calibration in the FrOST study. The scans covered the mid T12 vertebra to the mid L3 vertebra.

The paraspinal muscle and SAT volumes of interest (VOIs) were automatically segmented for L1 and L2 as illustrated in Fig. 1. Special care was taken not to include skin in the SAT VOI. The image analysis was performed using MIAF-Spine (ver. 6.0.5; MIAF: Medical Image Analysis Framework, University of Erlangen-Nürnberg, Germany). In addition, the TotalSegmentator (https://github.com/wasserth/TotalSegmentator) was employed to automatically segment the entire abdominal SAT at the L1/L2 levels, including anterior and peripheral SAT. CT values of paraspinal muscle and SAT VOIs were corrected for the water offset determined using the measured CT value of the water insert of the Osteo calibration phantom.

### 2.5 Effect of edema on CT values of SAT

**Fig. 3:**
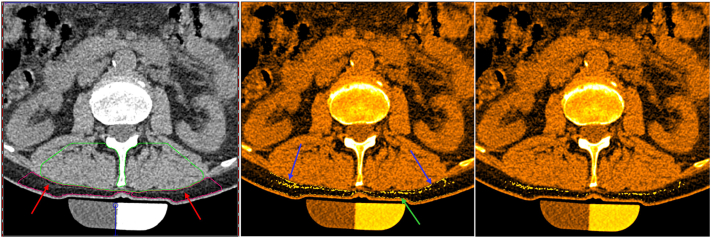
Left: CT with window and level adjusted to visualize edema (red arrows), which has higher CT values than SAT. Center: Edema voxels identified by the algorithm based on the skew of the distribution of all CT voxels of the SAT VOI are shown in yellow (blue arrows) in the false color image. Due to partial volume artifacts voxels of the border of the SAT VOI (green arrow) were also labelled as edema. Right: After peeling the SAT VOI by two voxel this effect was largely reduced.

**Fig. 4:**
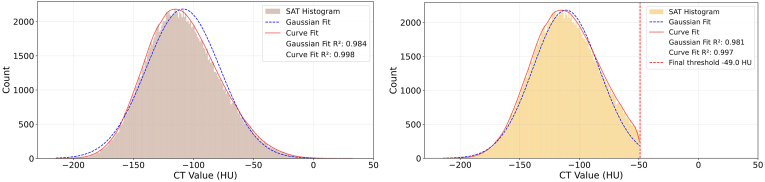
SAT Histogram analysis of the CT dataset from Fig. 3. Left: Histogram of the SAT CT values before correction. Mean −101.7 HU, skew 0.34. The skew was calculated from the red curve. The blue curve shows a fitted Gaussian. Right: Histogram after iterative removal of voxels with higher CT values as described in the text. In this example the voxels with CT values > −47 HU were removed, corresponding to 4.1% of all voxels. ‘Corrected’ mean of −104 HU, ‘corrected’ skew −0.0002.

SAT often includes fluid retention known as edema. Edema has X-ray absorption characteristics similar to water resulting in CT values close to zero. Larger agglomerations of edema are visible in CT images with appropriate windowing (Fig. 3 left). Assuming that SAT is a homogeneous material and that its noise distribution is Gaussian, the presence of edema would skew the shape of the histogram of the CT values of the SAT VOI (Fig. 4). Therefore, identifying and subsequently removing the voxels responsible for the skew - with the aim of restoring a Gaussian shape to the histogram of the remaining voxels - should reduce the effect of edema on SAT CT values used for the muscle tissue density calibration. An empirical target skew of 0.0025 was selected to prevent overcorrection while preserving the underlying SAT distribution.

The skew of the histogram derived from all SAT VOI voxels was calculated using SciPy (version 1.10.1; https://scipy.org/). Mean and standard deviation (SD) were also calculated from the SAT histogram. In a first step, all voxels with a CT value threshold T greater than the mean plus 4 SD were removed for a skew greater than 0.0025. Subsequently, a new histogram and its skew were computed. T was then decreased iteratively by 1 HU, and voxels with CT values greater than T were eliminated. This process was repeated until the target skew of 0.0025 was reached. A comparison was made between the original and processed SAT distributions. The comparison was based on descriptive statistics, which included the mean HU values, SDs, and change in skewness.

As can be seen in Fig. 3, some of the voxels labelled as edema by the above procedure are located at the border of the SAT VOI. Therefore, in a final step, these voxels were excluded by peeling the SAT VOI by two voxels.

### 2.6 Accuracy errors of the proposed muscle calibration model

Accuracy errors were simulated by changing the default values of the following parameters, which were used for the muscle calibration: SMT mass density (1.05 g/cm^3^), SMT CT value (48 HU), FF of SMT (3%), FF of SAT and IMAT (85%), mass density of AT (0.95 g/cm^3^) and water offset correction (phantom-based value). Compared to a CT value of −100 HU corresponding to a FF of SAT of 85%, a FF of 82% (88%) corresponds to a CT value of −94.5 HU (−105.5 HU). The results for ρ_MT_, ρ_M_ and FF were averaged over all 42 scans. Deviations from mean ρ_MT_, ρ_M_ and FF resulting from the change in default values were compared to results based on the default values using paired T-tests.

### 2.7 Comparison of muscle fat fraction between CT and MRI Dixon sequences

In the FrOST study MRI scans of the paraspinal muscles were also obtained (3T MAGNETOM Skyra-fit, Siemens) [22]. Specifically, a T1 water-weighted turbo spin echo (T1w) sequence (voxel size: 0.4 × 0.4 × 3 mm^3^) and a 6 pt Dixon sequence (voxel size: 0.8 × 0.8 × 3 mm^3^) of the paraspinal muscles of L2 to L4 were available for 31 of the 41 participants included in the current study. Fat fraction maps that directly code the percentage of fat of each voxel were calculated from the acquired Dixon sequences. This is a standard procedure of the MRI scanner software [3]. MRI scans were obtained one to two months after the CT scans were performed [22].

For the purposes of this study, the T1w images were manually segmented using ImageJ (version 1.51). To ensure consistency between the MRI and CT analyses, the segmented CT images were displayed alongside the T1w images and then 10-12 consecutive axial slices covering L2 were segmented matching the paraspinal VOIs of the CT images as close as possible (Fig. A4). The segmentation masks of the T1w images were transferred to the corresponding FF maps using 3D rigid registration [23]. Finally, the FF results were compared between the MR and CT images.

## 3 Results

### 3.1 CT values of muscle and adipose tissue

The results of the water offset corrected phantom measurements are shown in Table 1. The mean SMT CT value, averaged over all scanners and tube voltage settings, was 47.7 ± 1.9 HU. There was a significant (one-way ANOVA, p = 0.02) tube voltage related decrease of 4.4 HU in the mean CT value, from 49.9 HU at 80 kV to 45.5 HU at 140 kV, reflecting the slight difference of the mass absorption coefficients of SMT and water. The water offset corrections were less than 2 HU for the X-cite, go. Top and ICT-SP scanners and less than 5 HU for the Force, Optima 67 and Aquilion Prime scanners. For the Icon Spectral scanner, the water offset corrections were −11 HU at 80 kV, −8 HU at 100 kV, - 6 HU at 120 kV and −3 HU at 140 kV.

**Table 1:** CT values [HU] for ICRU muscle tissue/adipose tissue for 80 - 140 kV. CT values after water offset correction. The first shows CT manufacturers and type of scanner. SI: Siemens, PI: Philips, GE: General Electric; TO: Toshiba. ∗ GE results were invalid at 80 kV due to severe artifacts in BDC phantom.

	SI X.cite	SI go.Top	SI Force	PI ICT-SP	PI Icon Spectral	GE Optima 67	TO Aquilion Prime	Mean ± SD
**CT values muscle [HU**]								
80 kV	47.3	48.2	49.4	45.7	50	∗	52.9	**49.9 ± 2.5**
100 kV			46.5		50	47.6	49.2	**48.3 ± 1.6**
120 kV			45.5		48	45.3	49.1	**47.0 ± 1.9**
140 kV	45.2	44.4	44.4	45.3	46	45.9	47.7	**45.5 ± 1.2**
**Mean ± SD**	**46.3** ± **2.7**	**46.2** ± **1.5**	**46.5** ± **2.2**	**45.5** ± **0.3**	**49.5** ± **1.9**	**46.3** ± **1.2**	**49.7** ± **2.2**	**47.7 ± 1.9**
**CT values adipose tissue [HU**]								
80 kV	−101.2	−99.6	−96.0	−94.2	−99.5	−88.2	−93.7	**−96.1 ± 3.1**
100 kV			−83.7		−77.7	−70.9	−77.2	**−77.4 ± 5.2**
120 kV			−75.7		−74.2	−62.6	−73.3	**−71.5 ±6.0**
140 kV	−71.8	−72.1	−71.4	−66.0	−69.4	−55.9	−72.1	**−68.2 ± 5.8**
**Mean ± SD**	**−85.7** ± **19.6**	**−86.6** ± **20.6**	**−81.7** ± **10.8**	**−80.1** ± **19.9**	**−80.2** ± **13.3**	**−63.1** ± **7.5**	**−78.8** ± **10.2**	**−76.8 ± 12.4**

The largest deviation from the mean SMT CT value of 47.7 HU was observed for the Aquilion Prime scanner at 80 kV with 5.2 HU. Considering only the tube voltages of 100 kV or 120 kV, which are most commonly used in the spine, the mean SMT CT was 47.7 ± 0.9 HU for the 4 scanners, for which measurements were taken, i.e. the mean CT value was identical to the mean for all 4 tube voltages, but the SD was smaller. At 100 kV or 120 kV the largest deviation from the mean SMT CT value was observed for the GE scanner at 120 kV. For a given tube voltage, the largest deviation from the corresponding mean of 4.2 HU was observed for the Philips ICT at 80 kV.

As expected, the results for AT, also shown in Table 1 show a large significant decrease of 28 HU, or of more than 25% between 80 and 140 kV.

### 3.2 Effect of edema on CT values of SAT

L1 and L2 were analyzed in 35 CT scans. In four CT scans L1 and in three CT scans L2 could not be analyzed. In each CT dataset, the results were averaged over all analyzed vertebrae. The distributions of SAT CT values from 41 CT scans were skewed towards larger CT values. One CT dataset was found to have a negative skew of −0.203, no correction was made, and this dataset was not included in the edema correction results summarized in Table 2. On average, the mean CT value of the SAT VOI after water correction was −97.4 HU. The minimum and maximum values were approximately 10 HU higher or lower than the mean. The mean SD of the distribution of SAT CT values was 28.9 HU. The average skewness was 0.27 ± 0.11 with minimum and maximum values of 0.03 and 0.50, respectively.

**Table 2:** Exclusion of edema voxels, 41 datasets used for analysis. Upper 4 rows: SAT results before correction. Lower 5 rows SAT results after correction. SAT: subcutaneous adipose tissue, SD: standard deviation, HU Threshold: all voxels of the SAT VOI with CT values > threshold were removed. ∗significant difference (p < 0.001) between before and after correction. Mean, Min and Max values are given for the 41 subjects.

	Mean ± SD	Min	Max
SAT Mean [HU]	−97.4 ± 4.57	−104.4	−84.9
SAT SD	28.9 ± 1.14	26.9	31.3
Water offset correction [HU]	−2.51 ± 1.29	−5.83	0.39
Skew	0.270 ± 0.109	0.034	0.496
After removal of edema			
SAT Mean [HU]	−99.0∗ ± 4.66	−107.5	−86.8
SAT SD	26.9 ± 0.74	23.1	29.3
Δ SAT [HU]]	−1.65 ± 0.74	0.26	3.69
Skew	−0.003∗ ± 0.003	−0.011	0.002
Removal of voxels - HU threshold	−33.0 ± 8.52	−56	−18
% voxels removed	2.05 ± 1.03	0.35	5.73

After excluding CT values above the CT dataset-specific thresholds (mean threshold −33.0 ± 8.5 HU), the mean skewness was reduced to −0.003 ± 0.003, i.e. the SAT CT value distributions of all datasets were close to a Gaussian. The effect on SAT scores was small. On average, only 2.05 ± 1.03% of the voxels of the SAT VOI were labelled as edema and the 'corrected' average SAT value was −99.0 ± 4.7 HU compared to −97.4 ± 4.6 HU before. The mean difference was −1.65 ± 0.74 HU. The difference in skew and mean SAT CT values before and after correction was significant (paired t-tests, p < 0.001). Fig. 3, Fig. 4 show an example. Fig. 3 center visualizes the ‘edema voxels’, which in this case had CT values greater than −47. Δ SAT was 1.85 HU without and 1.87 with erosion, and the HU threshold for voxel removal was −31.1 without and −33.3 HU with erosion.

### 3.3 Calibration of CT scans and simulation of accuracy errors

Calibrated values according to the equations detailed in the Appendix were obtained for L2 only, as the MR data did not include L1. 3 CT scans did not cover L2, thus 39 CT scans were included in this analysis.

Results are shown in Table 3 starting in the third row with the default settings of the constants used in the calibration equations. For a given constant, the subsequent rows show the effect of assumed deviations from its default value on the results of ρ_MT_, ρ_M_ and FF. For comparison, the last two rows of Table 3 show the effect of a ±5 HU offset in water correction.

**Table 3:** Effects of assumed deviations from default values for constants a - e entering the calibration equations (see Appendix) on muscle tissue density ρ_MT_, muscle density ρ_M_ and FF. The default values are shown below. Each table row shows the isolated effect of a change of the default value for one constant. Empty fields: no effect of change in given constant. a: mass density of SMT default 1.05 g/cm^3^ b: CT value of SMT default 48 HU c: FF of SMT default 3% d: FF of SAT and IMAT default 85% e: mass density of AT default 0.95 g/cm^3^ f: Water correction offset default phantom based value^u^ not significant. difference among means (p > 0.1 univariate Anova)^v^ significant difference versus default settings (p < 0.001 paired T-test)^w^ significant difference among means (p < 0.001 univariate Anova).

	ρ_MT_ [g/cm^3^]				ρ_M_ [g/cm^3^]				FF [%]			
	Mean	SD	Min	Max	Mean	SD	Min	Max	Mean	SD	Min	Max
all default settings	0.94^u^	0.08	0.61	1.06	1.04^w^	0.01	1.01	1.05	11.7^u^	6.4	2.0	37.2
a = 1.03	0.92^u^	0.08	0.60	1.04	1.02^w^	0.01	1.00	1.03				
a = 1.07	0.96^u^	0.08	0.62	1.08	1.06^w^	0.01	1.02	1.07				
b = 45	0.96^u^	0.08	0.61	1.06	1.04^w^	0.01	1.01	1.05	10.1^u^	6.5	0.3	36.1
b = 50	0.93^u^	0.08	0.60	1.05	1.04^w^	0.01	1.01	1.05	12.7^u^	6.3	3.2	37.9
c = 5									13.4^v^	6.2	4.1	38.3
c = 8									16.1^v^	6.0	7.1	40.1
d = 82									11.4^u^	6.2	2.1	39.9
d = 88									12.0^u^	6.6	2.0	38.4
e = 0.92					1.03^w^	0.01	1.00	1.05				
e = 0.97					1.04^w^	0.01	1.02	1.05				
f = +5	0.97^v^	0.08	0.64	1.1	1.04^v^	0.01	1.01	1.05	9.06^v^	6.6	0	35.3
f = −5	0.91^v^	0.08	0.62	1.1	1.04^v^	0.01	1.01	1.05	14.1^v^	6.2	4.8	38.9

The effects on ρ_MT_ and ρ_M_ were less than 3% compared to the default settings. Variation of SMT mass density or SMT CT value using the values in the table had no significant effect on ρ_MT_ (p = 0.14 univariate Anova) but the effect on ρ_M_ was significant (p < 0.001). Accuracy errors caused by a ±5 HU water offset correction error were lower than 4% and higher than those caused by variations of the other constants.

In absolute terms, accuracy errors in FF caused by a ±5 HU water offset correction error were significant (p < 0.001) but smaller than 3%. Variations in SMT CT or SAT FF had no significant effect on FF. Increasing the assumed SMT FF from 3% to 5% significantly increased FF (p < 0.001 paired t-test) by up 2% in absolute terms but similar to changes in the other constants, effects on FF were still smaller than those caused by a 5 HU water calibration offset error. However, this was no longer the case when increasing the assumed SMT FF from 3% to 8%, which increased FF by up to 5% in absolute terms.

Replacing the scan specific CT value of SAT with the water offset corrected CT value of the EDP AT insert, which was −75.71 HU for the Somatom Force scanner at 120 kV, ρ_MT_ decreased by 0.02 g/cm^3^, corresponding to a decrease of 2%. Linear regression gave ρ_AT_ = 1.11 ∗ ρ_SAT_ - 0.18, r^2^ = 0.994, p < 0.001. Absolute FF increased by 1.43%. Linear regression gave FF_AT_ = 1.11 ∗ FF_SAT_ + 0.19, r^2^ = 0.994, p < 0.001. Using a FF of 75% instead of 85% for ICRU AT, the absolute FF increased by only 0.2%, a percentage increase of 1.89%.

As the calibration is a linear transformation, the correlations between paraspinal CT values and ρ_MT_ or ρ_M_ were very high; (r^2^ = 0.994, p < 0.01). CT values of the complete SAT VOI differed by −3.7 ± 5.7 HU (min −17.5 HU, max 9.5 HU) from those of the posterior SAT VOI shown in Fig. 1 but the effect on ρ_MT_ or ρ_M_ was less than 1%.

### 3.4 Comparison of muscle fat fraction between CT and MRI

**Fig. 5:**
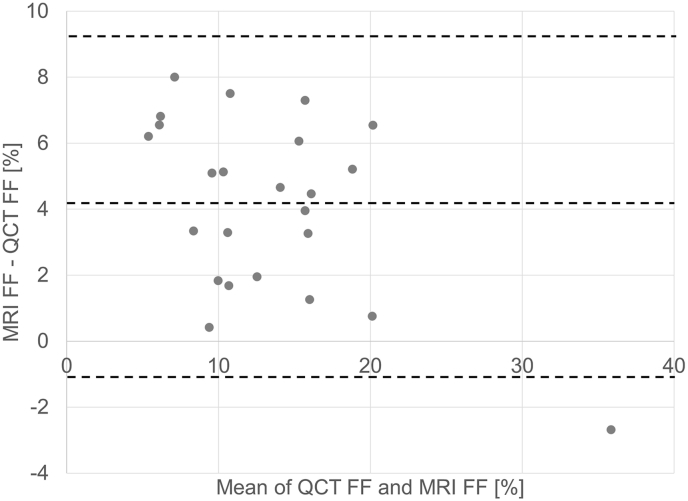
Blant Altman Plot showing MRI-CT FF differences versus MRI-CT FF mean values. Mean difference = 4. %. 95% LOA +9.34%; −1.1%.

24 of the 31 MRI datasets could be used to compare muscle FF. 7 MRI datasets were excluded due to image artifacts in the Dixon sequences affecting data quality or incomplete overlap of L2 between CT and MRI. The Bland-Altman plot in Fig. 5 shows that MRI FF values were on average 4.1% higher than CT FF values.

The regression equation was MR FF = 0.76 ∗ CT FF + 6.80. The results of the apparent outlier with a mean FF of 34% were carefully examined. MR and CT images did indeed show a high fat infiltration. When this high FF data point was excluded, r^2^ was 0.78 (p < 0.05). The regression equation was MR FF = 0.8 ∗ CT FF + 6.50. As expected, r^2^ of the linear regression of MRI FF versus CT FF was 0.88 (p < 0.001) identical to the r^2^ of the linear regression of the MRI FF versus CT values due to the linear relation between CT values of muscle and CT FF.

## 4 Discussion

In this proof-of-concept study, we introduced a new phantomless method to quantify muscle tissue density, muscle density and muscle FF on CT images. The proposed calibration technique is based on several reasonable assumptions but still needs to be discussed by the scientific community. This study was not intended to provide a comprehensive validation but rather to introduce a novel technique. However, an estimation of the impact of variations in the assumed values of the constants required by the calibration algorithm on accuracy errors, was performed.

The ICRU-defined adult skeletal muscle material [16,17] with a mass density of 1.05 mg/cm^3^, was selected as calibration reference on the assumption that this density (and higher densities) characterizes contractile muscle found in young healthy individuals. The ICRU based SMT definition is not sex and not muscle-specific although different muscles have different functions and even in young healthy individuals muscle tissue from different muscles may have different mass densities. Although potential implications for muscle calibration must be investigated, it is highly desirable to move towards a universally accepted muscle tissue standard. The ICRU SMT definition is particularly promising as phantoms containing long-time stable material compositions with equivalent densities and x-ray absorption characteristics, such as the EDP, already exist.

A key feature of the muscle calibration is the independence of the SMT CT value from scanner type and kV setting. Within the diagnostic range of 80–140 kV the difference in linear absorption coefficients between SMT and water remains fairly constant (Fig. A4). Consequently, and as confirmed in this study, the CT values of SMT were very similar for different scanners. Tube voltage related significant differences of up to 5.2 HU were observed within the 80 to 140 kV range. However, if accuracy errors of up to 5 HU are acceptable, a typical value if a water offset correction is not performed, a CT value of 48 HU can be used for SMT. Otherwise, scanner and kV specific CT values are recommended. It is important to note that differences related to tube voltage were not significant in the range from 100 to 120 kV, which is usually used for paraspinal, abdominal and thigh muscle measurements. Thus, a value of 48 HU could be used in multicenter studies using a large variety of CT scanners of different models and from different manufacturers.

SAT was used as the reference material to define a ρ_MT_ of 0 g/cm^3^ (Fig. 2). SAT largely consists of AT. Apart from edema the composition of SAT is comparable across different subjects but as demonstrated by the EDP phantom measurements, the CT value of AT varies with X-ray tube voltage. There is no kV-independent standard for AT or SAT. Therefore, the CT value of SAT was measured directly in the CT scan to be calibrated.

The Muscle calibration is a linear process (Fig. 2). As confirmed in the calibrated CT data, ρ_MT_, ρ_M_ and FF correlate highly with the muscle CT value (r^2^ > 0.99). Calibrated muscle parameters are measured in g/cm^3^. For the first time, the muscle mass in g or kg can be determined once the muscle volume has been measured. This is important because until now muscle mass could not be measured using any imaging technique.

Calibrating the CT value of water to 0 HU (and the CT value of air to −1000 HU) at any tube voltage is a key feature of all CT scanners to ensure comparability of soft tissue contrasts. However, in clinical routine small instabilities of a CT scanner often result in water CT values that deviate slightly from 0 HU, even if the scanner was well calibrated a few hours before the scan. The scans used for this study included a bone mineral density phantom containing a water insert that actually showed water offset values between +1 and −5 HU, thus the ±5 HU range was used as a benchmark. Such a CT calibration offset caused relative errors in ρ_MT_ and ρ_M_ and absolute errors in FF of less than 5%. Similar accuracy errors in FF have been reported when comparing MRI to MR spectroscopy [24,25].

Accuracy errors in ρ_MT_, ρ_M_ and FF which can be caused by measurement errors in CT values as well as by incorrect values used for the constants used in the calibration equations, namely mass density and FF of SMT and FF of SAT. This study investigated the effect of variations in these values on data from the FrOST study (Table 3). Relative errors in % were used for ρ_MT_ and ρ_M_. For FF, which is measured in % absolute errors were used, because FF was below 3% in five subjects of the CT data set. Thus, an absolute accuracy error of 1.5% would result in a relative error of 50%, severely limiting the value of using percentage change in FF.

Accuracy errors in ρ_MT_ due to measurement errors in CT values of muscle (Fig. A2) or SAT (Fig. A3) are small for ρ_MT_ > 0.8-0.9 g/cm^3^ but increase rapidly for smaller ρ_MT_, highlighting the importance of studies in cohorts with low muscle density. Errors in the measured CT values may be caused by inaccurate segmentation of the muscle or SAT VOI, by inaccurate exclusion of edema and fibrotic tissue in the SAT VOI (which was not an issue in the current study) or by an inadequate correction of the water offset. Based on the results of this study, the potential errors in the measurements of the CT values of muscle and SAT were below 5 HU.

Several constants are required for calibration. For AT mass density, the ICRU value of 0.95 g/cm^3^ was used. SMT FF was determined using 6 pt Dixon imaging in the thigh muscles of young healthy subjects, which were assumed to be representative of SMT. In elderly subjects, the FF of muscle tissue increases but for the calibration FF of SMT is required. The measured FF value of 3% was consistent with values reported in other publications [24,26,27]. For SAT published FF values for adipose tissue of 85% were used [14,15]. FF of SAT is age independent [14] and was found to change by only 1% after weight loss in obese subjects [28].

Reasonable ranges of values consistent with the results of those publications were used to simulate accuracy errors relative to the use of the assumed default values. Although a true accuracy gold standard of accuracy based on appropriate phantoms or tissue samples is required, it is reassuring that reasonable variations in the values used for the constants did not exceed the ±5 HU error caused by an inaccurate water offset correction.

It is interesting that the effect of inaccurate scanner calibration has so far been completely neglected in CT-based muscle research. Even a 5 HU error would cause a 10% accuracy error in a muscle density measurement of 50 HU using CT values, with an even larger percentage error for lower densities.

A special situation is edema, for example in subjects with lipidemia [29]. Edema artificially increases the CT values of SAT. Segmentation of edema in CT images is challenging, ‘mainly because of unclear boundaries with surrounding adipose tissue varying intensity distribution, and irregular shapes’ [30,31]. In this study, a newly developed method based on the skewness of the histogram of the SAT CT values was successfully used to identify and subsequently remove SAT voxels with higher CT values. In the subjects of the FrOST study, the effect of edema on the SAT CT value was negligible but this may differ in subjects with larger amounts of subcutaneous edema. However, the development of this method was incidental to the study. Its main purpose was to demonstrate that an edema corrected SAT CT value can be used to calibrate muscle tissue density.

The suggested ‘edema removal’ method may not be effective in other cohorts. In this case, an alternative internal method must be developed or a phantom with an AT standard, such as the EDP, must be scanned asynchronously to determine the CT value of SAT for the purpose of muscle calibration. However, in this case, the phantom must be scanned on the same scanner and with the same tube voltage as the subject scans. As a side remark: 'edema voxels' should not be excluded when measuring SAT volume, an important parameter in conditions such as obesity.

It is important to differentiate between whole organ muscle and muscle tissue, and therefore between ρ_MT_ and ρ_M_. The mass density of SMT is 1.05 mg/cm^3^. In the study population of elderly men with osteosarcopenia, average ρ_MT_ (i.e. the concentration of muscle tissue within the paraspinal muscle) was 0.94 g/cm^3^. On average, 90.6% of the paraspinal muscle volume was muscle tissue, with the remainder being adipose tissue (with a mass density of 0.9 g/cm^3^). Taken together, this resulted in an average paraspinal ρ_M_.of 1.04 g/cm^3^. With increasing age, the contractile muscle is replaced by IMAT, resulting in a decrease in ρ_MT_ and ρ_M_. In myopathies, certain muscles are completely replaced by adipose tissue. A muscle CT value of 48 HU corresponds to a muscle tissue density of 100% (1.07 g/cm^3^) and a ρ_M_ of 1.04 g/cm^3^ (0.9 g/cm^3^).

The method's ability to perform phantom-less calibration is an important benefit, as most CT scans used for muscle imaging do not contain a calibration phantom. Muscle imaging is also becoming increasingly important in opportunistic CT, which involves using existing CT scans obtained for other medical reasons. However, these scans often involve the application of CT contrast agents. A recent study found that a dose of 500 mg iodine per kg body weight (Imeron® 350; Bracco Imaging GmbH, Germany) increased CT values of paraspinal muscle by up to 7 HU [32]. Slightly higher values were reported in another study [33]. The muscle calibration method introduced in this study does not include any corrections for CT value enhancements related to contrast agents. Accurate correction may only be possible with dual-energy CT.

In principle, the calibration method introduced in this study is phantomless. However, a retrospective analysis of muscle tissue density is only possible in CT scans without contrast and if the CT value of subcutaneous adipose tissue (SAT) can be used, i.e. if it is corrected for potentially large amounts of edema that cause changes in SAT CT values of more than 10 Hounsfield units (HU). If phantom-based CT values must be used for adipose tissue, then the calibration is no longer internal and a retrospective muscle density analysis becomes more difficult. As shown in Table 1 CT values for adipose tissue are scanner-specific and depend on tube voltage. Therefore, using phantom scans acquired after the subject scans to be analyzed may not be possible, e.g. if the scanner used for the subject scans is no longer available. Additionally, the longitudinal stability of the scanner required to apply current phantom results to previously acquired scans may be questionable. So far, there is also no solution for determining the water offset correction in internal calibration without a phantom.

The first application of muscle calibration to a cohort of elderly men with osteosarcopenia showed good, albeit imperfect, agreement between CT and MR fat fraction (FF). Of the FrOST study subjects, all but one had a paraspinal FF of less than 20%, with MR FF averaging 4.1% higher than CT FF in these subjects. Further improvement of the results could probably be achieved by registering the CT and Dixon images in 3D to better align the analysis VOIs, which was not performed in this study. However, even if the 4.1% bias could be corrected, there would still be absolute differences of up tp ±4% between the MRI and CT FF. These differences may in part be explained by the time lag of one or two months between CT and MR acquisitions. Further, only a few studies reported on the accuracy data of Dixon FF. Differences between Dixon sequences and between Dixon and MR spectroscopy were in the order of a few percent have been reported [19,34].

There are several further limitations. The phantom measurements presented in Table 1 were not repeated. The density results obtained in the CT images were not compared to an external standard, such as a muscle biopsy. The results need to be confirmed in larger cohorts and in subjects with higher FF corresponding to lower ρ_MT_. Thus, validation in women, in patients with muscle disease and at other anatomical sites, such as the thigh are required. Errors in CT precision need to be determined and accuracy errors investigated in more detail, although this study has addressed several sources of accuracy error. The accuracy of the MRI results should also be revisited. The impact of intracellular water must be determined. The CT and MRI datasets available for the study were small, and the technique used to correct the CT values of SAT for edema may be problematic in other datasets. However, most items on this to-do list were beyond the scope of a proof-of-concept study. The 'edema removal' technique worked in the CT dataset of this study but could be replaced if it does not work in general.

For example, we investigated using −75.7 HU instead of specific SAT CT values. −75.7 HU was the CT value of the ICRU-specified AT material measured on the Siemens Force scanner at 120 kV, which was used in the FrOST study. It remains unclear whether this CT value, which was approximately 20 HU higher than the average SAT CT value of −97.4 HU, can be explained by the ICRU AT material having a lower FF. However, the most interesting finding was the extremely high correlation (r^2^ > 0.99) of the muscle FF results in the FrOST study when using the fixed value of −75.7 HU compared to using the SAT CT values. It is of great interest to establish whether this may allow the use of a scanner- and tube voltage-specific, but otherwise scan-unspecific, CT value, as this would eliminate the need to remove edema from SAT.

In conclusion, this study proposes a new method for determining muscle tissue density, muscle density and muscle fat fraction from measurements of CT values in muscle and subcutaneous adipose tissue (SAT) in single-energy CT images. Calibrating CT values to ρ_MT_, ρ_M_ and FF does not require a phantom, but rather the definition of standardized muscle tissue with a known mass density. Such a material has been defined by the ICRU. Using a CT value of 48 HU for this material, independent of the CT scanner or tube voltage, resulted in maximum relative accuracy errors of less than 5% for densities and absolute accuracy errors of less than 5% for FF. There was good agreement between MRI and CT FF values. On average, MRI FF values were 4.1% higher than CT FF values, with a high linear correlation. The preliminary results obtained in this study for the paraspinal muscles require further validation.

## CRediT author statement

**Jonathan Bammessel:** Methodology, Software, Validation, Formal analysis, Writing – Original Draft, Writing – Review & Editing.

**Stefan Bartenschlager**: Methodology, Software, Validation, Investigation, Formal analysis, Writing– Review & Editing.

**Oliver Chaudry:** Software, Data Curation, Investigation, Formal analysis, Writing– Review & Editing, Visualization.

**Nicolai Krekiehn:** Investigation, Writing– Review & Editing.

**Fjola Johannesdottir:** Conceptualization, Methodology, Formal analysis, Writing– Review & Editing.

**Ling Wang:** Conceptualization, Methodology, Validation, Writing– Review & Editing.

**Michael Uder:** Resources.

**Georg Schett:** Resources, Supervision.

**Klaus Engelke:** Conceptualization, Methodology, Validation, Formal Analysis, Writing – Original Draft, Writing, Writing– Review & Editing, Supervision, Funding acquisition.

## Declaration of AI and AI-assisted technologies in the writing process

The open source software TotalSegmentator (https://github.com/wasserth/TotalSegmentator) was employed to segment SAT. TotalSegmentator uses AI based techniques for automatic segmentation of CT datasets.

## Funding

This study was in part supported by the Bundesministerium für Bildung und Forschung (10.13039/501100002347BMBF) Project ARTEMIS - Artificial intelligence musculoskeletal disorders study (reference [01E]C190B).

## Declaration of competing interest

The authors declare that the research was conducted in the absence of any commercial or financial relationships that could be construed as a potential conflict of interest. KE is a part-time employee of Clario, Inc.
