# Supplementary material for: Quantification of skeletal muscle density, mass and fat fraction using single-energy computed tomography
Source: J Orthop Translat. 2026 May 7;58:101094. doi: 10.1016/j.jot.2026.101094 (PMC13185934; doi:10.1016/j.jot.2026.101094)
Supplement: Multimedia component 1 [file mmc1.docx]

Quantification of Skeletal Muscle Density, Mass and Fat Fraction using Single-Energy Computed Tomography Supplement

The calibration starts with the determination of the amount of standard muscle tissue (SMT, mass density 1.05 g/cm^3^) equivalent material, i.e of the muscle tissue density ρ_MT_ within the muscle. If the muscle consists of 50% MT and 50% IMAT then obviously ρ_MT_ = 1.05 / 2 = 0.525 g/cm^3^. ρ_MT_ is determined from a linear calibration (Figure 2). If in general a is the mass density and b the CT value of SMT, the resulting equation is

$\rho_{MT}=\frac{a}{-{CT}_{SAT}+b} {CT}_{muscle}+ a\left( 1- \frac{b}{-{CT}_{SAT}+b} \right)= \frac{a}{-{CT}_{SAT}+b}\left( {CT}_{muscle}-{CT}_{SAT} \right)$ ,

where CT_SAT_ and CT_muscle_ are the CT values of the SAT and muscle VOIs of a given subject.

In the CT value interval [CT_SAT_ ; b] the percentage of IMAT in the paraspinal muscle (%IMAT) can be calculated as

%$IMAT=-100\left( \frac{{CT}_{muscle}-b}{-{CT}_{SAT}+b} \right) =-100 (\frac{\rho_{MT}}{a}-1)$

If c is the muscle FF in % and d is the SAT FF in % the paraspinal FF can be determined from the measurement of the muscle CT and the SAT CT values as follows

$$FF=\left( \frac{d-c}{{CT}_{SAT}-a} \right) {CT}_{Muscle}+c-\left( \frac{a\left( d-c \right)}{{CT}_{SAT}-a} \right)=\left( \frac{d-c}{{CT}_{SAT}-a} \right) \left( {CT}_{Muscle}-a \right)+c$$

Finally using a mass density of 0.95 g/cm^3^ for adipose tissue, the muscle density ρ_M_ in g/cm^3^ is determined as

$$\rho_{M}=\frac{\%IMAT}{100}*0.95+\left( 1-\frac{\%IMAT}{100} \right)*1.05$$

If the muscle volume is known then muscle mass and muscle tissue mass, both in g or kg can also be calculated from ρ_M_ and ρ_MT_, respectively.

As explained in the main text in this study a FF of 3% for the ICRU reference muscle and a SAT FF of 85%, was assumed. Figure A1 and Figure A2 allow to calculate the % error of ρ_MT_ due to measurement errors of either the CT value of muscle or of SAT assuming for simplicity that the other measurement is accurate. For combinations of errors the first formula above can be used. The error in the SAT CT value may also be caused by a deviation of the fat fraction from the assumed value of 85%. A resulting difference of 10 HU corresponds to a lower FF of 79.5% potential caused by an increase in edema. A special case is a missing or inadequate correction of the CT water offset value, that affects CT values of muscle and SAT the same, that is the resulting % error in ρ_MT_ only depends on the effect on CT_SAT_ but in contrast to the results shown in Figure A 2 and Figure A 3 not on ρ_MT_. An error in the water correction of 5, 10 or 15 HU results in % error in ρ_MT_ of 3.5%, 7,2% or 11.3% respectively.

1. ICRU. *ICRU Report 44: Tissue Substitutes in Radiation Dosimetry and Measurement*. 1989 [cited os23.

2. ICRU. *ICRU Report 46: Photon, Electron. Proton and Neutron Interaction Data for Body Tissues*. 1992 [cited os24.

## Figures


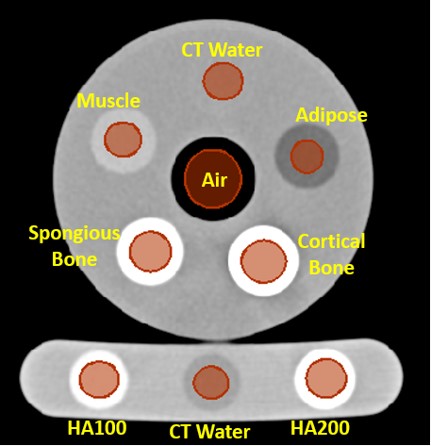


Figure A 1: CT image of electron density phantom (EDP) on top of bone density calibration phantom (BDC). The EDP contains tissue composition as specified by the ICRU [[1](#_ENREF_1), [2](#_ENREF_2)]. The brown circles show the segmentation of inserts. They are smaller than the inserts to avoid any partial volume artifacts of the measurement of the CT values. For this study the muscle and adipose tissues inserts were used. The CT value measured in the water insert of the BDC phantom was used for a water offset correction.


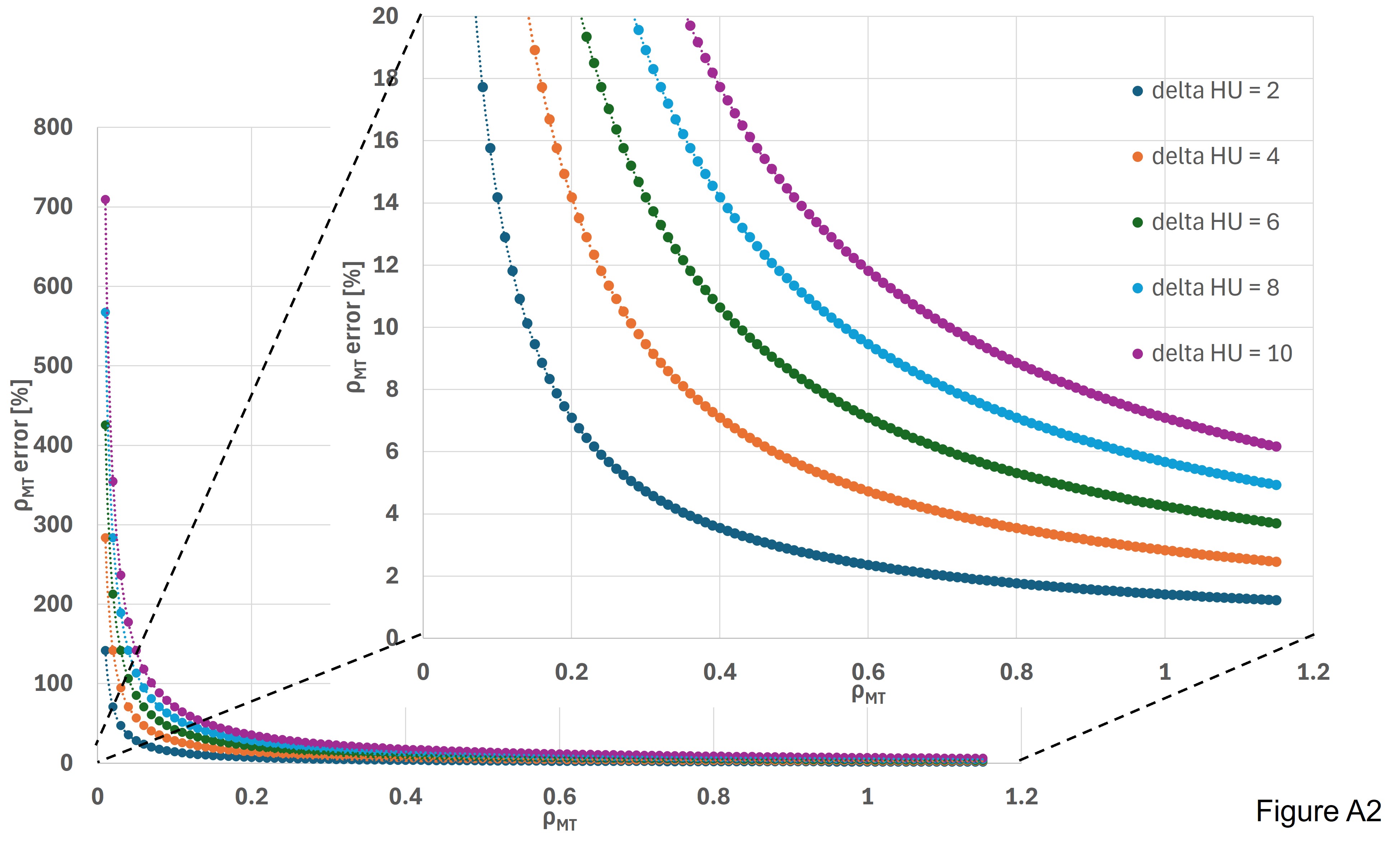


Figure A 2: % error in muscle tissue density ρ_MT_ caused by inaccuracies in the muscle CT value. The graph shows results for CT value inaccuracies of 2, 4, 6, 8 and 10 HU. The SAT CT value was set
 to -100 HU. The cutout shows the same data limited to a %error range of 0 to 20%.


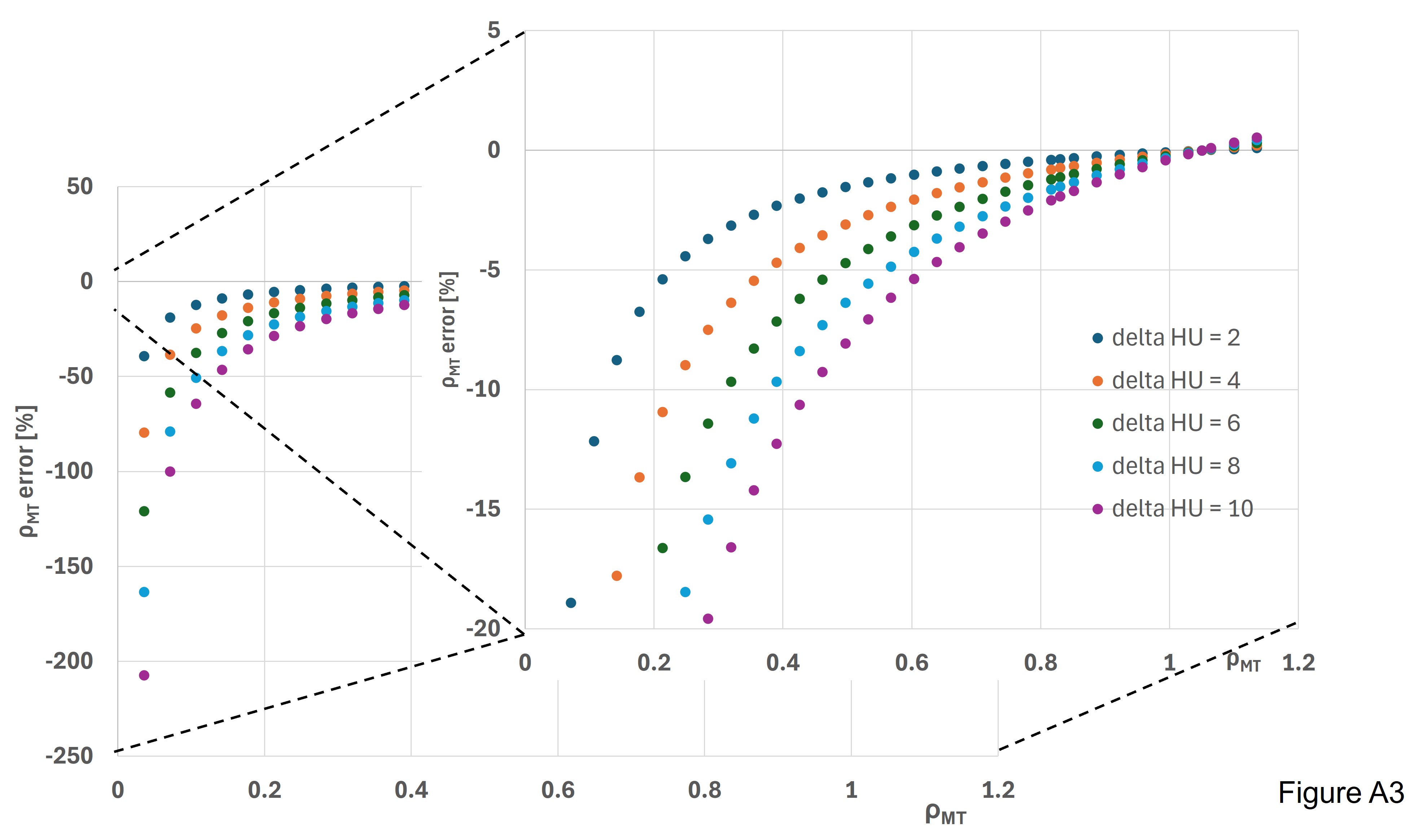


Figure A 3: % error in muscle tissue ρ_MT_ caused by inaccuracies in the SAT CT value. The graph shows results for CT value inaccuracies of 2, 4, 6, 8 and 10 HU. The default SAT CT value was set to -100 HU. The cutout shows the same data limited to a %error range of 5 to -20%.


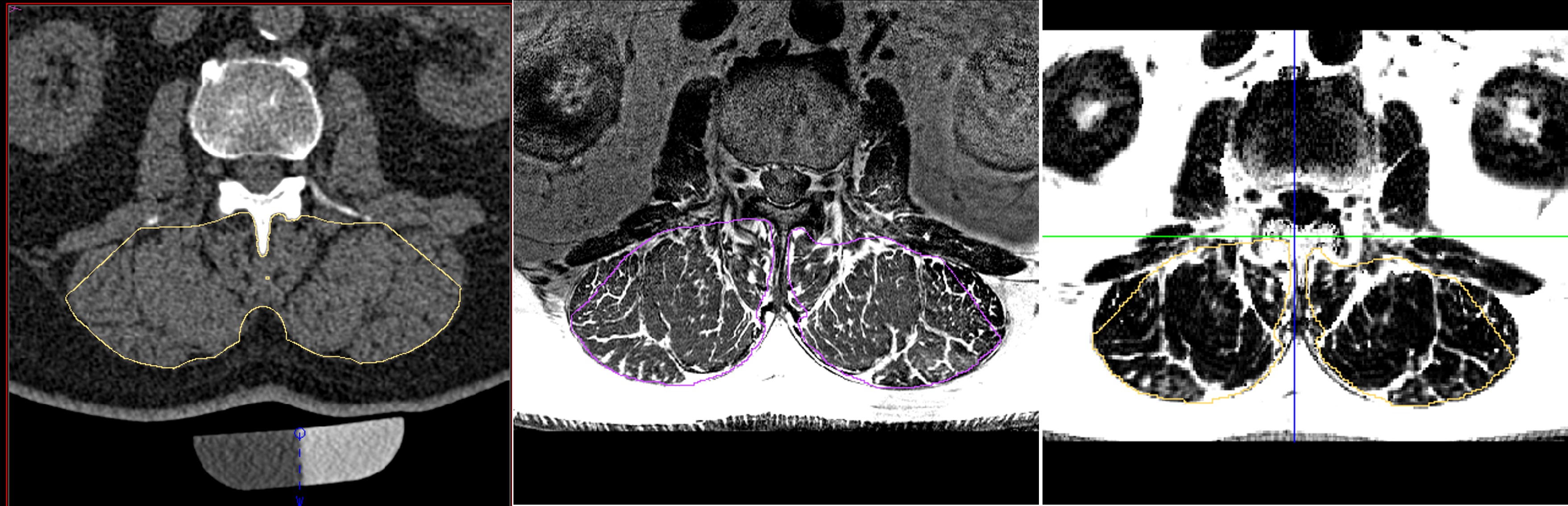
Figure A 4: Comparison of CT and MRI images showing one slice of L2. Matching segmentation of paraspinal muscles. Left: CT image; center: T1 weighted image used for segmentation; right: Dixon map to measure fat fraction after 3D registration to T1 weighted image of paraspinal VOI. Registration between CT and MR images was not performed


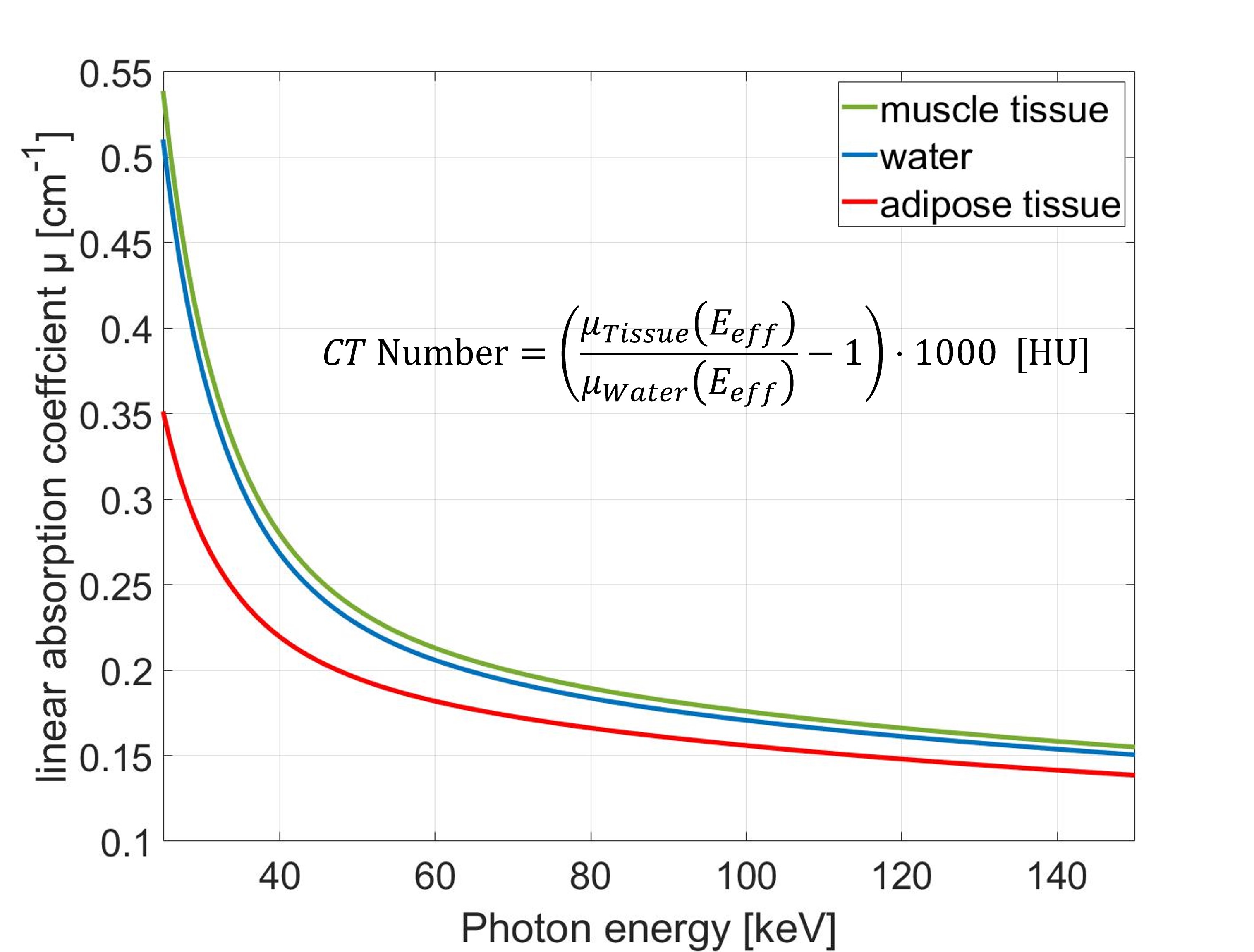


Figure A 5: linear absorption coefficients of water, muscle tissue and bone. Per definition for each CT scanner and tube voltage the CT values is calibrated to 0.
